# Organic mulches reduce crop attack by sweetpotato weevil (*Cylas formicarius*)

**DOI:** 10.1038/s41598-019-50521-5

**Published:** 2019-10-16

**Authors:** Mudassir Rehman, Jian Liu, Anne C. Johnson, Taiwo Esther Dada, Geoff M. Gurr

**Affiliations:** 1 School of Agricultural and Wine Sciences, Charles Sturt University, PO Box 883, Orange, NSW 2800 Australia; 2 Graham Centre for Agricultural Innovation (Charles Sturt University and NSW Department of Primary Industries), Wagga Wagga, 2650 New South Wales, Australia; 3 State Key Laboratory of Ecological Pest Control for Fujian and Taiwan Crops, Institute of Applied Ecology, Fujian Agriculture and Forestry University, Fuzhou, 350002 China; 4 Joint International Research Laboratory of Ecological Pest Control, Ministry of Education, Fuzhou, 350002 China; 5 Fujian-Taiwan Joint Innovation Centre for Ecological Control of Crop Pests, Fujian Agriculture and Forestry University, Fuzhou, 350002 China

**Keywords:** Ecosystem services, Plant ecology

## Abstract

Mulching with organic materials is a management practice with long history for weed suppression, soil water conservation and erosion control. Its potential impact on crop pests is less well explored. Here we report its utility for reducing crop damage by the serious pest, sweetpotato weevil (*Cylas formicarius*). Laboratory bioassays measured the response of adult female weevils to sweetpotato storage roots beneath mulches of fresh or dried plant materials. Weevils were significant repelled by fresh basil, catnip, basil lime and dry eucalyptus, cypress, lucerne and sugarcane. A subsequent field study found that mulches of dry cypress, eucalyptus and lucerne reduced movement of weevils from a release point to reach sweetpotato plants and lowered level of damage to storage roots. Results demonstrate that mulching with organic materials merits further testing as part of the integrated management of sweetpotato weevil, particularly to protect developing storage roots during dry periods when soil cracking can facilitate access by pests.

## Introduction

Mulches are defined as organic or synthetic materials that are applied as a cover to the soil surface and are widely used in various agricultural systems^1^. Mulches can suppress weeds, conserve soil moisture and reduce erosion but those consisting of organic materials offer an additional range of potential benefits. These include enhancing soil organic matter and associated soil biological activity, soil nutrient status, and moderated soil temperature^1,2^. Much less well explored is the potential of mulches to contribute to pest suppression via phenomena such as constituting a physical barrier to pest access to vulnerable crop parts, or making host plant detection more difficult by virtue of the chemical composition and volatile production by mulch material^3^.

Sweetpotato weevil, *Cylas formicarius* (Coleoptera: Apionidae), is globally the most destructive pest of sweetpotato, *Ipomoea batatas*^4,5^. Adult *C. formicarius* feed on sweetpotato foliage and the larvae damage stems but the most serious impact results from larval attack to the storage roots that are the harvested commodity. Direct feeding damage to the storage roots is compounded by the plant producing defensive sesquiterpenes that make the roots strongly distasteful, as well as by promoting damage by fungi and bacteria^6^. Losses to weevils are especially severe under dry seasons when soil cracking makes access to the roots easier for gravid females^7^. Storage roots may also be exposed to pest attack when their swelling, possibly combined with soil erosion from rainfall, leads to the cover of soil being breached. Sweetpotato weevil adults have limited capacity to dig through the soil^8^ so, in the absence of these conditions, they can reach storage roots only with difficulty. Though *C. formicarius* adults are known to fly, dispersal is chiefly via the use of infected planting material or by adult weevils walking from infested crops to newly planted nearby areas^9^. Accordingly, preventing the initial movement of *C. formicarius* into a crop and minimising subsequent access to storage roots are key to managing this pest^4,10^.

Insecticides, the use of pathogen tested planting material, and trapping with sex pheromones are used with success in sweetpotato productions systems in developed countries but are less available or affordable in developing countries where it is necessary to develop low-cost approaches^4,11^, potentially including pest-deterring mulches of locally-available materials. Even developed countries may benefit from the availability of new methods to lessen reliance on costly inputs and slow the development of insecticide resistance. Mulches have previously been used in sweetpotato cropping to suppress weeds, provide nitrogen, minimise soil erosion, encourage beneficial insects^11,12^ and to reduce pest attack^7^.

The aim of the present study was to explore a range of organic mulch treatments to determine their potential utility for sweetpotato weevil management, specifically by preventing their movement through layers of mulch and reducing levels of infestation achieved in storage roots. Initial laboratory screening of mulch treatments were designed to address two scenarios of field use by farmers. First, the application of mulch over partially exposed storage roots with the control treatment consisting of storage root covered by potting mix. This control reflected the farmer manually cultivating the field soil to repair cracks whilst the mulch treatments represented the less laborious application of mulch to cracked areas. The second study compared mulches with a control in which the storage root remained partially exposed. This reflected the farmer not cultivating to repair cracked soil and compared the mulch treatments with this ‘no-action’ scenario. A smaller number of mulch treatments was subsequently evaluated in small field plots, again with covered and uncovered storage root fragments to address both management scenarios.

## Methods

### Insect and plant materials

*Cylas formicarius* were collected from farms in Cudgen, New South Wales and Bundaberg Queensland as immatures within infested storage roots and supplemented by adult males caught using sex-pheromone traps. A laboratory colony was maintained at the Orange campus of Charles Sturt University (33.2465°S, 149.1173°E) using growth chambers ran at 26 °C ± 2 °C, 60 ± 5%RH, 12:12L:D photoperiod. Insects were supplied with supermarket-purchased sweetpotato storage roots that were replaced on a weekly basis. Infested sweetpotatoes were kept in separate containers to give specific cohorts of emerging adults. Females were discriminated from males based on antennal morphology and only these were used in experiments. Preliminary studies identified a need to move adult females from the high density rearing/emergence vessels for a period of 24 hours in lower density vessels in order for them to exhibit biologically faithful responses to various plant stimuli. Without this measure, females exhibited very strong dispersal irrespective of external conditions. Accordingly we use a protocol that took cohorts of females 10–15 days after eclosion and held these under low density conditions without food for 24 h at 26 °C ± 2 °C prior to use in all studies.

The mulch materials used were selected based on a literature review that indicated their likely production of compounds with activity against herbivore Coleoptera^13–18^. Each was either (i) a potential secondary crops that could be cultivated in conjunction with sweetpotato so that crop residues or excess plants (e.g., thinnings, prunings) could be used as mulch or (ii) organic materials that are likely to be cheaply available or readily produced locally.

Potential secondary crops that were tested as freshly chopped fragments were: spring onion (*Allium fistulosum* L.), basil (*Ocimum basilicum* L.), catnip (*Nepeta cataria* L.), chilli (*Capsicum annuum* L), lime basil (*Ocimum americanum*, L.), tobacco (*Nicotiana tabacum* L.), oregano (*Origanum vulgare* L.), French lavender (*Lavandula stoechas* L.), white onion (*Allium cepa* L.), lemongrass (*Cymbopogon citratus* (DC.) Stapf), marigold (*Tagetes patula* L.), Mexican sunflower (*Tithonia rotundifolia* (Mill.) S.F. Blake) and spearmint (*Mentha spicata* L.). Dried mulch materials were: sugarcane (*Saccharum officinarum* L.), lucerne (*Medicago sativa* L.), wheat straw (*Triticum aestivum* L.), eucalyptus (*Eucalyptus albens* Benth.) and cypress (*Cupressus* × *leylandii* A. B. Jacks. & Dallim).

Fresh materials were purchased as live plants from a plant nursery (Thompson’s Garden Centre, Orange, New South Wales). The mulch was prepared by chopping all aboveground plant parts into 2–3 cm long pieces with clean scissors. Dry mulch materials, lucerne (Oreco Group, Organic Lucerne Mulch), sugarcane (Oreco Group, Sweet Garden Organic Sugar Cane Mulch), eucalyptus (ANL, Eucy Mulch) and cypress (Ki-Carma, Cypress Mulch) were purchased from Bunnings Warehouse (Orange, New South Wales) and used directly from the pack in their original, proprietary form. Wheat straw was purchased as a bale from Mullion Produce (Orange, New South Wales). An additional treatment of whole fresh cabbage leaves was included in the field experiment and this used freshly-collected plants from the University farm.

### Laboratory screening of mulches

The mulch materials were divided into groups for testing: Group A (spring onion, sugarcane and lucerne); Group B (basil, catnip and chilli); Group C (lime basil, tobacco and oregano); Group D (French lavender, white onion and lemongrass); Group E (marigold, Mexican sunflower and spearmint); Group F (wheat straw, eucalyptus and cypress).

The initial screening of the mulch materials was carried out in multiple-choice test mesocosms made with plastic plant pots, 31 cm in diameter, and 26 cm in depth. The pots were half -filled with proprietary potting mix (Osmocote Professional Premium Plus Potting Mix, from Bunnings Warehouse, Orange, New South Wales), then a 90 mm diameter Petri dish base placed centrally on the potting mix surface of each. The area surrounding the Petri dish was then divided into four, equal sized quadrants. In the centre of each quadrant, a 50 g (±10 g) piece of sweetpotato storage root was positioned on the potting mix surface. This was then covered by one of the mulch materials, with the fourth quadrant as control. The first type of control left the root fragment uncovered to simulate exposure of storage roots in cracked field soil. The second type of control covered the root fragment with 2 cm of potting mix to approximate field conditions in which soil cover had been retained over developing storage roots.

Each group was replicated 3 or 4 times with the uncovered control and 3 or 4 times with the covered control. Mulches were applied to a 2 cm depth. Forty naive adult female *C. formicarius* were placed in each central Petri dish at around 3 pm and allowed to disperse into each of the equidistant mulch treatments. A Fluon (INSECT-A-STOP, Queensland) barrier was applied to the top edge of pots to prevented escape from mesocosms. After 24 hours, each piece of sweetpotato storage root was visually inspected and the number of weevils and feeding holes were recorded.

### Field testing of mulches

**Figure 1:**
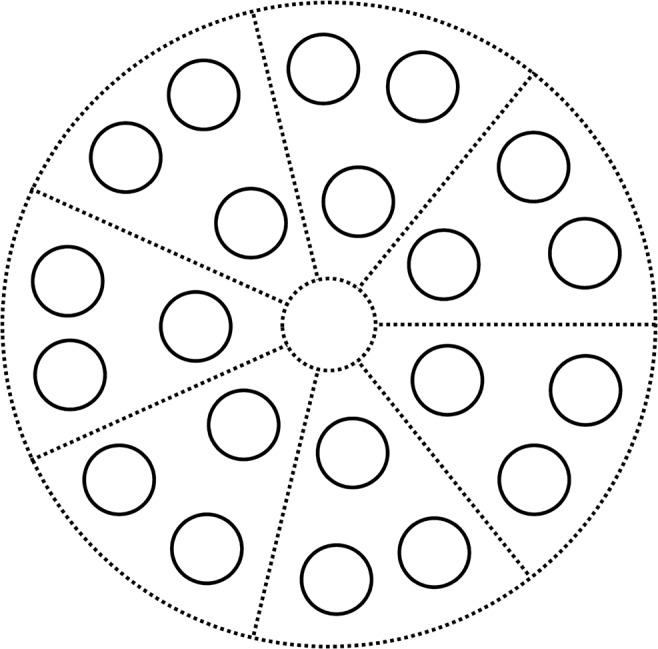
Illustration of multiple-choice test field design. Each block was 2 m in diameter with a central release point for weevils, surrounded by one plot of each mulch treatment. Plots each contained three sweetpotato plants illustrated in solid circles, one in the inner ring and two in the outer ring.

Mulch materials of lucerne, sugarcane, eucalyptus, cypress, wheat straw and cabbage leaves were selected for field testing. An area of grassland on the university campus farm, which does not have sweetpotato weevil, was cultivated to prepare four round blocks, each 2 metres in diameter and 2 metres apart. Each block was surrounded by a 60-cm-tall black plastic barrier (Whites Recycled Garden Edging), the base of which was sunk into the soil to prevent passage of weevils. Each of the four blocks was divided into 7 equal-sized wedges-shaped plots, with the central area as the weevils release point (Fig. 1). Within each plot, three  soil mounds, each 15 cm-tall and 25 cm in dimeter, were formed using hand tools. Accordingly, each block had 21 mounds with seven forming a ring in the inner part of the block and equidistant form the weevil release site. The remaining 14 mounds made up an outer ring (Fig. 1). Sweetpotato plants were transplanted singly into each mound at the end of January 2018. The control plot in each block had bare soil. Thereafter, plots were watered and hand weeded twice a week. Six weeks later, shop-purchased sweetpotato storage roots were selected with comparable size (each 200 ± 50 gram) and placed in plots. At this time, mulch materials were applied, one treatment to each plot, to a depth of 3 to 5 cm. A piece was placed either side of the inner sweetpotato plant, one fully covered by the existing mulch and a second piece sunk into the mulch but with the upper surface exposed. In the outer part of each plot, the two pieces of storage root were positioned equidistant between the two sweetpotato plants, one fully covered and one partly exposed. This method was adopted to assess the effects of mulching on two types of behaviour by the weevil: (i) lateral movement over the mulch surface to reach uncovered storage root, and (ii) vertical movement through the mulch layer to reach storage root covered by mulch after travelling laterally. In the control treatment where no mulch material was applied, field soil was used to partially or fully cover the storage roots. The mulch materials were given 24 h to settle before 140 adult female *C. formicarius* were released into the centre of each block.

*Initial Assessment* was done two days after weevil release. Sweetpotato foliage was visually inspected and the number of weevils present on the leaves and stems of each plant were recorded. All storage roots were visually inspected for weevils, then removed to the laboratory for inspection of feeding holes.

Immediately after the initial assessment, two new pieces of storage root were placed in each plot, one beside the sweetpotato plant in the inner ring and one between the two sweetpotato plants on the outer ring. These were fully covered with mulch except in the control treatment (no mulch) where storage roots were left uncovered.

*Second Assessment* was conducted 10 days after the weevil release. Sweetpotato foliage and storage roots were inspected for the presence of weevils a second time. The storage root pieces were removed to be inspected for feeding holes in the laboratory.

### Statistical analyses

Analysis of variance (ANOVA) and LSD test were conducted to compare the effect of mulch treatments on number of weevils reaching the sweetpotato and number of feeding holes using software IBM-SPSS. In the laboratory studies, the mesocosms of each control type were analysed separately. In the field study, a GLM univariate analysis of variance was conducted on the influence of three independent variables, mulch treatment, and distance from the central release area of weevils and whether or not the storage root fragment was covered. Figures were generated using Microsoft 2013 excel and package ggplot2 in R version 3.4.4^19^.

## Results

### Laboratory screening of mulches

**Figure 2:**
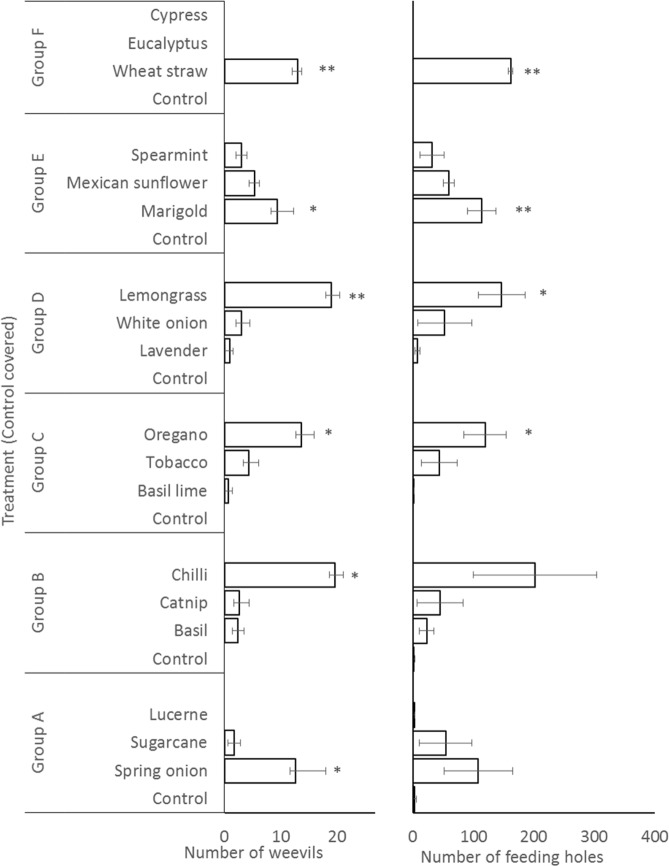
Effect of mulch materials in protecting sweetpotato storage root (Control covered by potting mix) (laboratory screening of mulches). (*means p < 0.05 when compared to control within the group; **means p < 0.01 when compared to control within the group).

**Figure 3:**
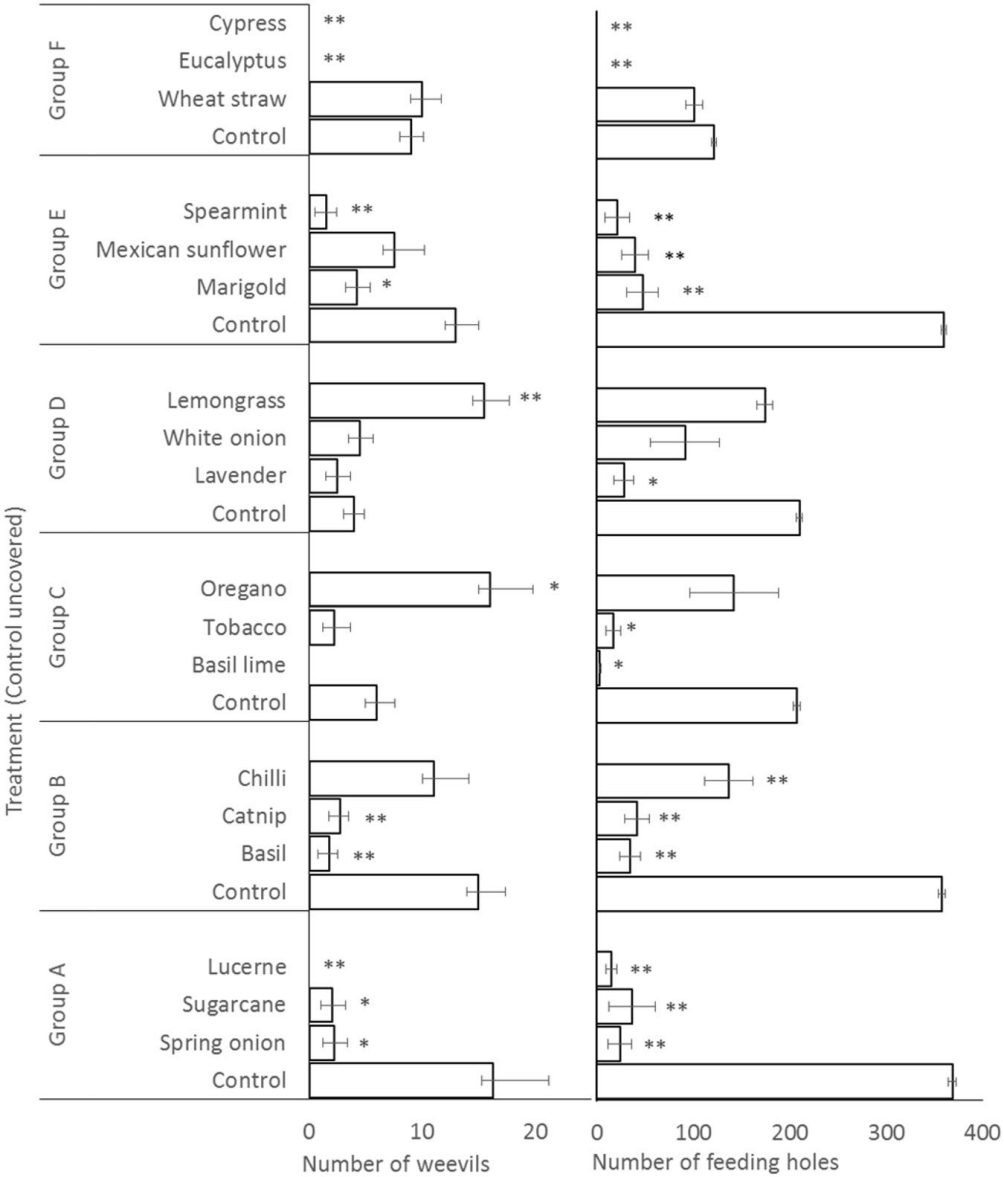
Effect of mulch materials in protecting sweetpotato storage root (Control uncovered by potting mix) (laboratory screening of mulches). (*means p < 0.05 when compared to control within the group; ** means p < 0.01 when compared to control within the group).

Experimental treatments had large and statistically significant effects on numbers of *C. formicarius* and incidence of their feeding holes on sweetpotato storage root pieces. Weevil numbers were low (0 been observed) in the control consisting of root pieces covered by potting mix, and significantly elevated, to >9 weevils per root fragment, by spring onion, chilli, oregano, lemon grass, marigold and wheat straw (Fig. 2). In the study in which the control had uncovered storage root pieces, relatively large numbers of weevils (between 4 and 16) were present on the storage root pieces and significant reductions (to <3) were evident in treatments of spring onion, sugarcane, lucerne, basil, catnip, basil lime, spearmint, eucalyptus and cypress (Fig. 3).

Numbers of weevil feeding holes on sweetpotato storage root fragments followed a broadly similar trend to the foregoing results for weevil numbers. In the covered root control (Fig. 2), <5 holes were recorded per root fragment but this rose significantly, to >100, in treatments of spring onion, chilli, oregano, lemongrass, marigold and wheat straw. This demonstrated that no mulch treatments gave weevil control that was more effective than covering storage roots with soil and that some mulches exacerbated damage.

For the study comparing mulches with an exposed fragment of storage root, numbers of feeding holes were high in the control, falling between 121 and 369. In contrast, significant reductions, less than 48 feeding holes, were evident for mulch treatments spring onion, sugarcane, lucerne, basil, catnip, basil lime, tobacco, lavender, marigold, Mexican sunflower, spearmint, eucalyptus and cypress. This demonstrates that a majority of the mulches tested gave significant levels of protection to exposed storage roots compared with a scenario of the farmer not having the time or labour to manually cover the exposed roots with soil.

In the first group of mulches when the piece of sweetpotato was left uncovered a significantly higher number of weevils and feeding holes were found on the control (no mulch) treatment. However, when the sweetpotato piece was buried in the potting mix the piece of sweetpotato under the spring onion was the least successful in masking the sweetpotato and attracted the highest number of weevils. There was no differences in the number of feeding holes for any of the treatments spring onion, sugarcane, lucerne or control when the control was covered (Fig. 2).

### Field testing of mulches

**Figure 4:**
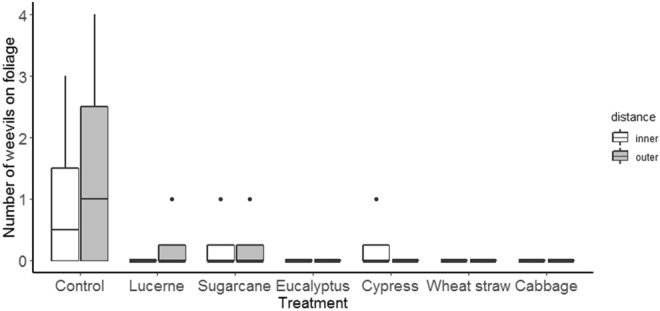
Effect of mulch on weevils’ movement towards sweetpotato foliage at initial assessment in the field test.

**Figure 5:**
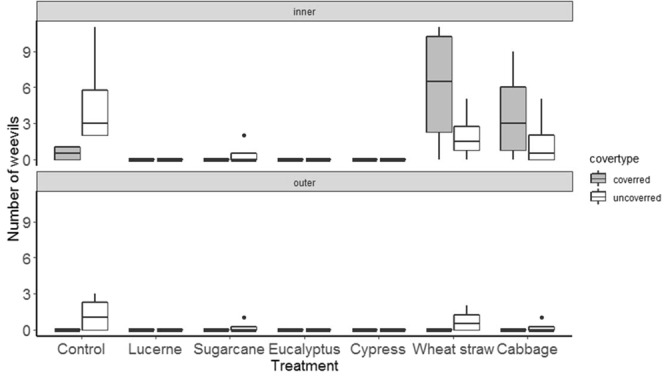
Effect of mulch on weevils’ movement towards sweetpotato storage root at initial assessment in the field test.

**Figure 6:**
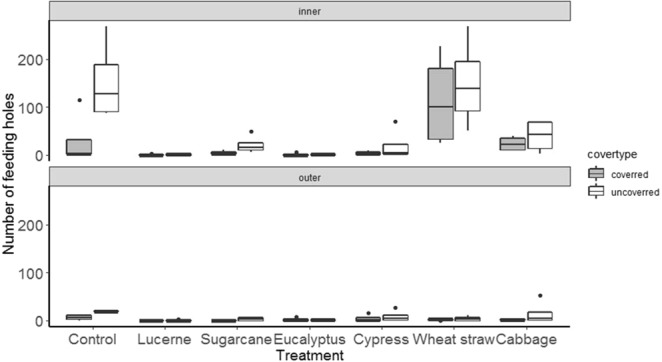
Effect of mulch on weevils’ feeding damage on sweetpotato storage root at initial assessment in the field test.

Reflecting the earlier laboratory results, mulches had strong effects on weevils under field conditions. Numbers of weevils on the foliage of sweetpotato plants was reduced by all mulch treatments, irrespective of the distance of plants from the central release zone (F_mulch_(6,42) = 3.4, p < 0.01; F_distance_(1,42) = 0.2, p < 0.7) (Fig. 4). This response variable did not, however, consistently agree with the more economically important variables of numbers of weevils reaching storage roots and levels of damage. Weevil numbers on storage root fragments were significantly affected by mulch treatment and distance (F_mulch_(6,84) = 4.9, p < 0.001; F_distance_(1,84) = 13.8, p < 0.001) with lower numbers in the outer distance and for the mulches lucerne, sugarcane, eucalyptus and cypress mulches (Fig. 5). For numbers of feeding holes, the same variables as well as whether the storage root was covered or not, all had significant effects ((F_mulch_(6,84) = 9.5, p < 0.001; F_distance_(1,84) = 29.8, p < 0.001; F_cover_ (1,84) = 7.2, p < 0.01). Numbers of holes in the inner distance were very much higher than in outer distance and here damage was greater in the control, wheat straw and cabbage treatments with damage also tending to be higher for unexposed storage root fragments (Fig. 6).

**Figure 7:**
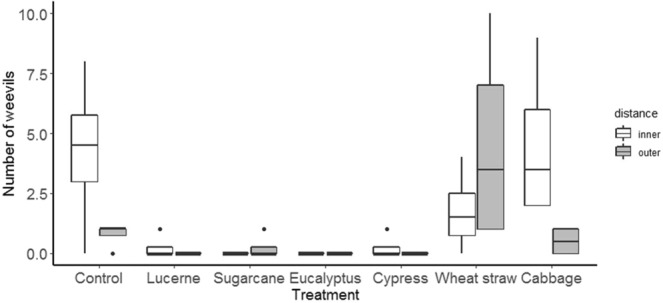
Effect of mulch on weevils’ movement towards sweetpotato storage root at 2^nd^ assessment in the field test.

**Figure 8:**
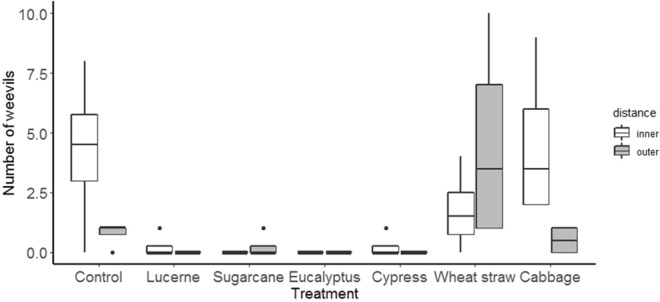
Effect of mulch on weevils’ feeding damage on sweetpotato storage root at 2^nd^ assessment in the field test.

For the second assessment, the weevils distribution was significantly affected by mulch treatment, with few present on storage roots covered by lucerne, sugarcane, eucalyptus or cypress mulches (F_mulch_(6,42) = 5.0,p < 0.01) (Fig. 7). Compared with the earlier assessment date, relatively large numbers of weevil feeding holes were evident on storage roots in the outer zone of plots. Notwithstanding this, feeding damage was significantly greater to storage roots in the centre part of each plot (F_distance_(1,42) = 12.5, p < 0.01) (Fig. 8). For this variable also, mulch treatment also had an effect (F_mulch_(6,42) = 4.4, p < 0.01) such that lucerne, eucalyptus and cypress mulches significantly reduced the number of weevils reached sweetpotato storage root compared to control treatment. Cabbage leaf and, especially, wheat straw and mulches afforded a poor level of protection to sweetpotato roots.

## Discussion

Mulches of 9 of the 18 types of plant material tested in the initial laboratory screen exhibited protective effects for sweetpotato against *C. formicarius*. Among the six treatments subsequently tested under field conditions, four provided significant levels of control of weevil colonisation of the crop and reduced feeding damage. This high incidence of biologically active plant species in both studies is of practical significance because it suggests that future testing of the plant materials that are readily and cheaply available to growers in a given region are likely to reveal a number of suitable candidate mulches with protective properties.

Mulches of synthetic material or of living or dead plant material are widely used in agriculture for a number of purposes, most commonly weed suppression and water conservation. This level of prior acceptance is likely to predispose farmers to be prepared to adopt mulches to serve a wider range of functions including pest management. In sweetpotato production, straw or plastic mulches have been used to reduce weed growth and extend the season in cooler climates^20^ and there has been one study suggesting utility against a congeneric of the species we studied, *C. puncticollis*^7^. In that African field study, freshly harvested, dried, chopped aerial parts of elephant grass (*Panicum maximum*) were applied to plots at 0, 1, 3 and 5t/ha. Mulching intensity was significantly and inversely associated with pest infestation rate of *C. puncticollis* and led to higher storage root yields. Though the present study did not include elephant grass, the apparent repellence of several of the mulches tested and reductions in feeding hole numbers are consistent with the effects reported by Mansaray *et al*.^7^. The Poaceae species tested in the present study, sugarcane, lemongrass and wheat straw, had inconsistent effects on *C. formicarius*, suggesting that taxonomic identity of the plant from which mulch material is sourced is likely to be less important as a predictor of efficacy than specific plant traits. Among the traits most likely to be key to efficacy in pest management is the chemical composition, particularly the production of volatiles. Indeed most of the plant materials showing effects in the present study were – to human perception – strongly smelling. Significantly, however, the diversity of plants that yielded efficacious mulches suggests that there is no single class of volatile compounds responsible for the observed effects on *C. formicarius*.

There is little information available on the chemistry of *Cupressus* × *leylandii*, the source of the cypress mulch that was effective in laboratory and field studies but it is anecdotally repellent to termites (http://www.cypressmulch.com.au/). Members of the genus reportedly have pinene- and -myrcene-rich terpeneoid volatiles^21,22^ that have widely-reported repellent effects on arthropods^23^ that are likely to be key to the effects observed on *C. formicarius*. Mulch of *Eucalyptus albens* was potent in laboratory and field tests and the members of this genus are known to produce volatile terpenes with activity against arthropod pests^24,25^. The sugarcane mulch tested in the present study was produced from leaves and tops rather than being the ‘bagasse’ by product of sugar processing. Though the plant nutrient composition of this material has been studied^26^ and insecticidal properties reported from lignin emulsion extracted from sugarcane waste^27^, there is no available information on the volatiles that are likely to be responsible for effects on *C. formicarius* in the present study. For lucerne hay, the fourth mulch material exhibiting potency in the present laboratory and field studies, a total of 147 peaks was reported in GC-MS studies of livestock feeding preferences^28^. That study did not seek to identify the compounds responsible for the GC-MS peaks and there is a lack of other studies of this topic. Among the species that were active in the laboratory studies, basil has previously been reported as useful suppressing other pests and is considered a useful companion plant due to the volatile is produces^29–31^. There is, however, no literature on its effects on pests when used as a mulch.

Sweetpotato weevil responses to volatiles are known to vary according to chemical composition^32,33^. The foregoing brief overview of the volatiles of the mulches with efficacy against *C. formicarius* illustrates that much further work needs to be conducted to establish a knowledge of the chemistry of mulches and to link this with results from behavioural studies of this species and other target pests of interest. Key to this is establishing the concentration and identity of volatile compounds in various types of mulches for this will affect the duration of effect after a mulch is applied. A short period of efficacy reflecting, for example, highly volatile active compounds, may not preclude utility in use against *C. formicarius* if application coincides with a period of high levels of crop protection such as during a drought when soil cracking becomes serious.

A wider range factors is likely to affect the net effect of mulches on pest management. Mulch application may lead to microclimatic changes within the crop canopy and to the soil as well as within the mulch itself and these could encourage natural enemies. In apples, for example mulch treatments including composted poultry manure led to lower soil temperature and higher soil moisture levels as well as an increase in edaphic detritivores and predators^28^. Cover crops that remain on soil surface after dying off, effectively resulting in a cover of mulch, increase the structural complexity of the soil surface and – combined with other factors – can lead to greater abundance of natural enemy species and lower abundance of pests^34^. The organic matter in mulches can enhance natural enemies independent of effects on physical structure and microclimate by promoting the densities of detritivore prey available to generalist predators, an effect that can translate into enhanced control of the focal pest is systems such as rice^35,36^. Cypress mulch has also been shown to promote predatory insects which in turn reduces insect pest incidence^37^. The addition of organic matter can also have a more general promoting effect on soil invertebrate biodiversity, for example sugarcane promoting ants and earthworms^38,39^. More widely, the presence of organic matter can have an effect on entomopathogens through protection from desiccation or ultraviolet light, an effect evident for the persistence of the entomopathogenic nematode, *Steinernema carpocapsae*, in plots of soybean^36^. Entomopathogenic nematodes of sweetpotato weevil are known^40^ therefore mulches could potentially be used to promote these. A further, potential beneficial effect of mulches is that the organic matter can promote entomopathogenic fungi by serving as substrate^41^. It is important to note that the foregoing, natural enemy-mediated effects were not operable in the present laboratory studies and were unlikely to have been significant in the subsequent field study. They do, however, need to be considered in future field evaluations.

A final set of factors that favour the potential value of organic mulches in sweetpotato production systems relate to broader agronomic issues. Stone *et al*.^42^ reported sweetpotato yields were promoted by a treatment involving a killed cover crop of vetch, with this likely to have resulted from the resulting mulch layer reducing soil temperature and promoting the development and bulking of storage roots^42–44^. Further, the decomposition of the organic matter in mulches adds nutrient to the soil^4,45,46^. Sweetpotato is not a good competitor of early season weeds^45^ and smallholder farmers often hand weed at this stage^47^, therefore mulches can help suppress weeds with the extra labour required to collect and apply the mulch offset by reduced need for labour to weed the crop.
